# Jewel beetles (Coleoptera, Buprestidae) collected during the 2018 field survey on Iejima Island, the Okinawa Islands, Ryukyus, south-western Japan

**DOI:** 10.3897/BDJ.8.e48785

**Published:** 2020-01-31

**Authors:** Yutaka Tamadera, Hiraku Yoshitake

**Affiliations:** 1 Laboratory of Entomology, Tokyo University of Agriculture, Atsugi, Japan Laboratory of Entomology, Tokyo University of Agriculture Atsugi Japan; 2 Kyushu Okinawa Agricultural Research Center (Itoman residence), NARO, Itoman, Japan Kyushu Okinawa Agricultural Research Center (Itoman residence), NARO Itoman Japan

**Keywords:** Buprestid beetle, fauna, distribution, new record

## Abstract

**Background:**

Only one jewel beetle, Chrysodema (Marcsikiella) dalmanni (Eschscholtz) (Coleoptera, Buprestidae) has hitherto been recorded from Iejima Is. in the Okinawa Isls., Ryukyus, south-western Japan.

**New information:**

A total of seven jewel beetles were collected on Iejima Is. The following six species are newly recorded from the island: 1) *Paratrachys princeps chujoi* Kurosawa, 2) Chrysodema (Chrysodema) lewisii Saunders, 3) *Chalcophora japonica oshimana* Schönfeldt, 4) *Coraebus hastanus* Gory and Laporte, 5) *Sambus quadricolor quadricolor* Saunders and 6) *Agrilus okinawensis shiozakii* Tôyama.

## Introduction

**Figure 1.**
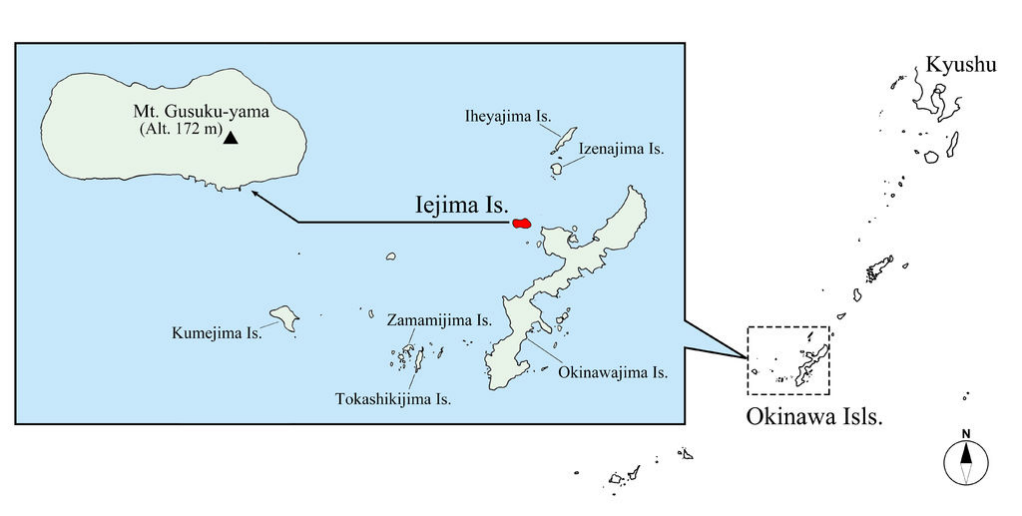
Location of the Okinawa Islands and Iejima Island in the Ryukyus, south-western Japan.

The Okinawa Islands in the Ryukyus, south-western Japan (Fig. 1) has one of the highest species diversity of jewel beetles (Coleoptera, Buprestidae) in Japan. From the islands, 44 buprestid species belonging to 18 genera in four subfamilies have hitherto been recorded, including five endemic species and five endemic subspecies (Ohmomo and Fukutomi 2013, Hattori 2014). This species number accounts for about 20% of all Japanese buprestid beetles. The buprestid fauna of the Okinawa Isls. has hitherto been well surveyed particularly in the largest island, Okinawajima Is. (area ca. 1,207 km^2^; 44 known species), while our knowledge on the fauna of the other islands is still limited due to the lack of sufficient surveys. For example, only eight species had been recorded from the second largest island, Kumejima Is. (area ca. 60 km^2^) until recently, when five more species were recorded by Tamadera et al. (2019).

**Figure 2.**
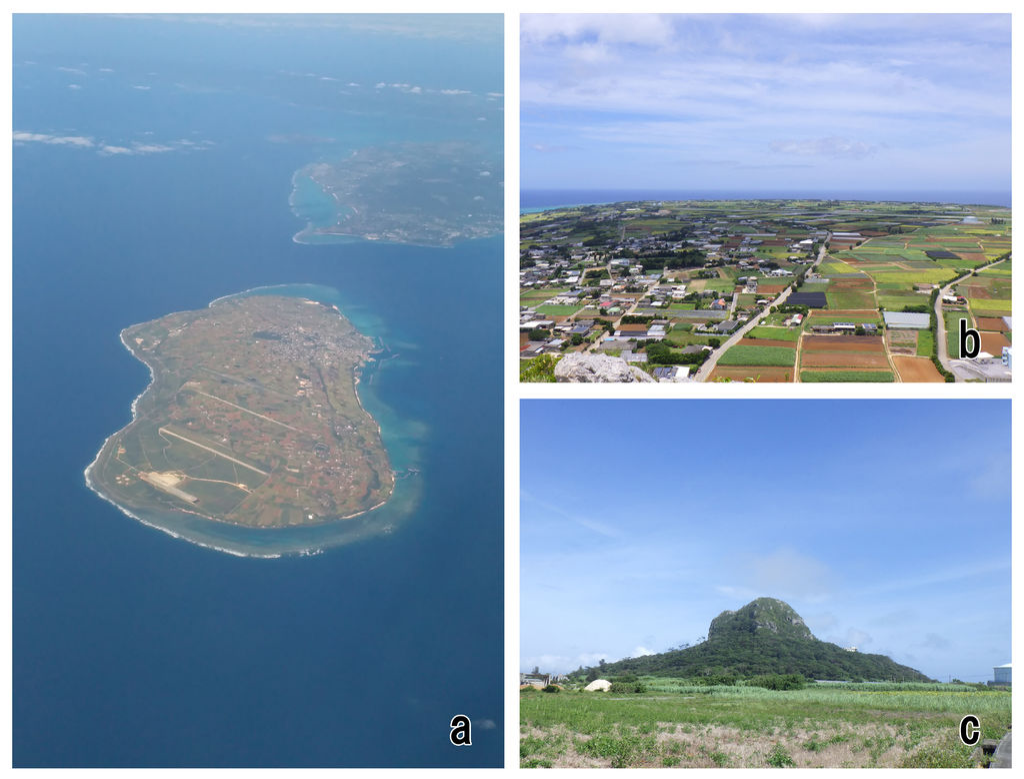
Iejima Island: **a**, Aerial view; **b**, View from the top of Mt. Gusuku-yama; **c**, Mt. Gusuku-yama.

Iejima Island, about 9 km off the north-western coast of the Motobu Peninsula in Okinawajima Is., is the third largest island (area ca. 22.8 km^2^) in the Okinawa Isls. (Fig. 1). Iejima Is. is mostly flat and covered with cropland, except a small evergreen broad-leaved forest is spreading in Mt. Gusuku-yama (altitude ca. 172 m), located in the central east (Figs 1, 2). Presently, only one buprestid beetle, Chrysodema (Marcsikiella) dalmanni (Eschscholtz, 1837), is known from the island (Kohama 2015, Yao 2016). However, more buprestids are expected to occur on the island, since no investigations on the buprestid fauna have been conducted until now. In June 2018, we conducted a field survey on Iejima Is. and collected seven buprestid beetles, six of which are new to the fauna of the island. In this paper, we will report them for the first time from Iejima Is.

## Materials and methods

Specimens used in this study were collected on Iejima Is. by the authors in a period from 8^th^ to 11^th^ June 2018. All the specimens were collected by net-sweeping or beating of plants, as well as by visual searching and then examined and identified by the first author (YT) under an Olympus SHZ10 stereomicroscope. In addition, associated plants with each buprestid species were identified by him. Scientific names of buprestid beetles followed recent catalogues edited by Löbl and Löbl (2016) and plant nomenclature followed Yonekura and Kajita (2003). Finally, all the specimens are preserved in the private collection of the first author.

A habitus photograph of each species was taken under a Canon 8000D digital camera. Each final image was assembled from a series of photographs with different focus planes, using the computer freeware CombineZP (Hadley 2010).

Abbreviations used for collector names in this study are as follows: HY — Hiraku Yoshitake; YT — Yutaka Tamadera.

## Taxon treatments

### Paratrachys princeps chujoi

Kurosawa, 1976

3FC6D481-68D3-5CCA-868B-2CC79DDC33D6

#### Materials

- **Type status:** Other material. **Occurrence:** recordedBy: Yutaka Tamadera; individualCount: 3; sex: unknown; lifeStage: adult; **Location:** islandGroup: Ryukyus; island: Iejima Island; country: Japan; stateProvince: Okinawa; locality: Wajii-tenbôdai, Higashie-ue, Ie-son; **Identification:** identifiedBy: Yutaka Tamadera; dateIdentified: 2018; **Event:** eventDate: 06/09/2018; **Record Level:** basisOfRecord: PreservedSpecimen

#### Distribution

Japan: the Okinawa Isls. (Okinawajima Is.; Iejima Is. — new record) and Yaeyama Isls. (Ishigakijima Is.) (Ohmomo and Fukutomi 2013).

#### Notes

New to Iejima Is., these specimens were collected by visual searching and beating *Ficus pumila* (Moraceae) which grows on a cliff facing the sea (Figs 3d, 4a).

### Chrysodema (Marcsikiella) dalmanni

(Eschscholtz, 1837)

C938EFC7-6F36-51B4-B318-0F5AA0120185

#### Materials

- **Type status:** Other material. **Occurrence:** recordedBy: Yutaka Tamadera; individualCount: 14; sex: 10 males, 4 females; lifeStage: adult; **Location:** islandGroup: Ryukyus; island: Iejima Island; country: Japan; stateProvince: Okinawa; locality: Ishara-hama, Higashie-mae, Ie-son; **Identification:** identifiedBy: Yutaka Tamadera; dateIdentified: 2018; **Event:** eventDate: 2018-06-10; **Record Level:** basisOfRecord: PreservedSpecimen
- **Type status:** Other material. **Occurrence:** recordedBy: Yutaka Tamadera; individualCount: 6; sex: 5 males, 1 female; lifeStage: adult; **Location:** islandGroup: Ryukyus; island: Iejima Island; country: Japan; stateProvince: Okinawa; locality: Ishara-hama, Higashie-mae, Ie-son; **Identification:** identifiedBy: Yutaka Tamadera; dateIdentified: 2018; **Event:** eventDate: 2018-06-11; **Record Level:** basisOfRecord: PreservedSpecimen
- **Type status:** Other material. **Occurrence:** recordedBy: Hiraku Yoshitake; individualCount: 6; sex: 4 males, 2 females; lifeStage: adult; **Location:** islandGroup: Ryukyus; island: Iejima Island; country: Japan; stateProvince: Okinawa; locality: Ishara-hama, Higashie-mae, Ie-son; **Identification:** identifiedBy: Yutaka Tamadera; dateIdentified: 2018; **Event:** eventDate: 2018-06-11; **Record Level:** basisOfRecord: PreservedSpecimen

#### Distribution

Japan: the Amami Isls. (Amami-Ôshima Is., Kikaijima Is. and Tokunoshima Is.), Okinawa Isls. (Iheyajima Is., Nohojima Is., Okinawajima Is., Hamahigajima Is., Zamamijima Is., Tokashikijima Is., Akajima Is., Fukajishima Is. andKumejima Is.; Iejima Is. — new record), Miyako Isls. (Miyakojima Is.) and Yaeyama Isls. (Ishigakijima Is., Iriomotejima Is., Kohamajima Is., Kuroshima Is., Haterumajima Is. and Yonagunijima Is.); Taiwan; Philippines; and Indonesia (Ohmomo and Fukutomi 2013, Aoki 2014, Kohama 2015, Inada et al. 2015, Yao 2016, Inada 2016, Sakai 2016b, Kusui and Miyagi 2017, Hinoki 2018, Tamadera et al. 2019).

#### Notes

These specimens were collected by net-sweeping *Terminalia catappa* (Combretaceae) (Figs 3e, 4b). In the collecting site, some adult exit holes of C. (M.) dalmanni were observed in thick dead branches of the plant.

### Chrysodema (Chrysodema) lewisii

Saunders, 1873

0745FCFB-D071-5085-974B-18739813C1DB

#### Materials

- **Type status:** Other material. **Occurrence:** recordedBy: Yutaka Tamadera; individualCount: 3; sex: 3 males; lifeStage: adult; **Location:** islandGroup: Ryukyus; island: Iejima Island; country: Japan; stateProvince: Okinawa; locality: Mt. Gusuku-yama, Higashie-ue, Ie-son; **Identification:** identifiedBy: Yutaka Tamadera; dateIdentified: 2018; **Event:** eventDate: 2018-06-08; **Record Level:** basisOfRecord: PreservedSpecimen
- **Type status:** Other material. **Occurrence:** recordedBy: Yutaka Tamadera; individualCount: 9; sex: 5 males, 4 females; lifeStage: adult; **Location:** islandGroup: Ryukyus; island: Iejima Island; country: Japan; stateProvince: Okinawa; locality: Mt. Gusuku-yama, Higashie-ue, Ie-son; **Identification:** identifiedBy: Yutaka Tamadera; dateIdentified: 2018; **Event:** eventDate: 2018-06-09; **Record Level:** basisOfRecord: PreservedSpecimen
- **Type status:** Other material. **Occurrence:** recordedBy: Yutaka Tamadera; individualCount: 1; sex: female; lifeStage: adult; **Location:** islandGroup: Ryukyus; island: Iejima Island; country: Japan; stateProvince: Okinawa; locality: Mt. Gusuku-yama, Higashie-ue, Ie-son; **Identification:** identifiedBy: Yutaka Tamadera; dateIdentified: 2018; **Event:** eventDate: 2018-06-10; **Record Level:** basisOfRecord: PreservedSpecimen
- **Type status:** Other material. **Occurrence:** recordedBy: Yutaka Tamadera; individualCount: 6; sex: 3 males, 3 females; lifeStage: adult; **Location:** islandGroup: Ryukyus; island: Iejima Island; country: Japan; stateProvince: Okinawa; locality: Mt. Gusuku-yama, Higashie-ue, Ie-son; **Identification:** identifiedBy: Yutaka Tamadera; dateIdentified: 2018; **Event:** eventDate: 2018-06-11; **Record Level:** basisOfRecord: PreservedSpecimen

#### Distribution

Japan: Honshu, Shikoku, Kyushu, the Izu Isls. (Hachijôjima Is.), Tsushima Is., Kuroshima Is. (in Mishima-mura), Ôsumi Isls. (Tanegashima Is., Yakushima Is. and Kuchinoerabujima Is.), Tokara Isls. (Nakanoshima Is. and Akusekijima Is.), Amami Isls. (Amami-Ôshima Is., Yorojima Is., Tokunoshima Is. and Okinoerabujima Is.), Okinawa Isls. (Iheyajima Is., Okinawajima Is., Tokashikijima Is., Zamamijima Is., Akajima Is., Gerumajima Is. and Kumejima Is.; Iejima Is. — new record), Miyako Isls. (Miyakojima Is.) and Yaeyama Isls. (Ishigakijima Is., Iriomotejima Is., Kuroshima Is. and Yonagunijima Is.); and Taiwan (Takai 1991, Ohmomo and Fukutomi 2013, Inada et al. 2015, Sakai 2016a, Sakai 2016b, Hinoki 2018).

#### Notes

New to Iejima Is., these specimens were collected by visual searching and net-sweeping *Elaeocarpus zollingeri* (Elaeocarpaceae) and *Toxicodendron succedaneum* (Anacardiaceae) (Fig. 3a, f). *Toxicodendron succedaneum* is not recorded as a host plant of C. (C.) lewisii, but this species may be associated with the plant at least during the adult stage, because some specimens were collected from it at several places.

### Chalcophora japonica oshimana

Schönfeldt, 1890

554DEF18-AC77-51C5-9CE3-6CDB8F462499

#### Materials

- **Type status:** Other material. **Occurrence:** recordedBy: Yutaka Tamadera; individualCount: 2; sex: 1 male, 1 female; lifeStage: adult; **Location:** islandGroup: Ryukyus; island: Iejima Island; country: Japan; stateProvince: Okinawa; locality: near Beigun-hojo-hikôjô-atochi, Nishie-ue, Ie-son; **Identification:** identifiedBy: Yutaka Tamadera; dateIdentified: 2018; **Event:** eventDate: 06/10/2018; **Record Level:** basisOfRecord: PreservedSpecimen
- **Type status:** Other material. **Occurrence:** recordedBy: Yutaka Tamadera; individualCount: 2; sex: 1 male, 1 female; lifeStage: adult; **Location:** islandGroup: Ryukyus; island: Iejima Island; country: Japan; stateProvince: Okinawa; locality: near Beigun-hojo-hikôjô-atochi, Nishie-ue, Ie-son; **Identification:** identifiedBy: Yutaka Tamadera; dateIdentified: 2018; **Event:** eventDate: 06/11/2018; **Record Level:** basisOfRecord: PreservedSpecimen

#### Distribution

Japan: the Amami Isls. (Amami-Ôshima Is., Kikaijima Is., Kakeromajima Is., Yorojima Is., Tokunoshima Is. and Okinoerabujima Is.), Okinawa Isls. (Iheyajima Is., Okinawajima Is., Agunijima Is., Zamamijima Is., Akajima Is., Gerumajima Is. and Fukajishima Is.; Iejima Is. — new record) and Miyako Isls. (Miyakojima Is.) (Ohmomo and Fukutomi 2013, Inada et al. 2015, Inada 2016, Sakai 2016b, Sakai 2017a).

#### Notes

New to Iejima Is., these specimens were collected by net-sweeping *Pinus luchuensis* (Pinaceae). The collecting site is a small forest left in cropland (Figs 4c, 5a).

### Coraebus hastanus

Gory and Laporte, 1839

27508B52-4E2D-568E-9B94-F6CD7EFF7E11

#### Materials

- **Type status:** Other material. **Occurrence:** recordedBy: Yutaka Tamadera; individualCount: 2; sex: 2 males; lifeStage: adult; **Location:** islandGroup: Ryukyus; island: Iejima Island; country: Japan; stateProvince: Okinawa; locality: Mt. Gusuku-yama, Higashie-ue, Ie-son; **Identification:** identifiedBy: Yutaka Tamadera; dateIdentified: 2018; **Event:** eventDate: 2018-06-09; **Record Level:** basisOfRecord: PreservedSpecimen
- **Type status:** Other material. **Occurrence:** recordedBy: Yutaka Tamadera; individualCount: 3; sex: 1 male, 2 females; lifeStage: adult; **Location:** islandGroup: Ryukyus; island: Iejima Island; country: Japan; stateProvince: Okinawa; locality: near Shôtaiji Temple, Nishie-mae, Ie-son; **Identification:** identifiedBy: Yutaka Tamadera; dateIdentified: 2018; **Event:** eventDate: 2018-06-10; **Record Level:** basisOfRecord: PreservedSpecimen
- **Type status:** Other material. **Occurrence:** recordedBy: Yutaka Tamadera; individualCount: 4; sex: 3 males, 1 female; lifeStage: adult; **Location:** islandGroup: Ryukyus; island: Iejima Island; country: Japan; stateProvince: Okinawa; locality: near Kawahira-kôminkan, Kawahira, Ie-son; **Identification:** identifiedBy: Yutaka Tamadera; dateIdentified: 2018; **Event:** eventDate: 2018-06-10; **Record Level:** basisOfRecord: PreservedSpecimen

#### Distribution

Japan: the Amami Isls. (Amami-Ôshima Is., Kikaijima Is., Tokunoshima Is. and Okinoerabujima Is.), Okinawa Isls. (Iheyajima Is., Izenajima Is., Okinawajima Is., Senagajima Is., Hamahigajima Is., Miyagijima Is., Tsukenjima Is., Yabuchijima Is. and Kumejima Is.; Iejima Is. — new record), Miyako Isls. (Miyakojima Is. and Ikemajima Is.) and Yaeyama Isls. (Ishigakijima Is., Iriomotejima Is. and Kuroshima Is.); Taiwan; China; Nepal; Bhutan; India; Burma; Thailand; Laos; Vietnam; Indonesia; and Philippines (Xu et al. 2013, Ohmomo and Fukutomi 2013, Kusui 2017, Kusui and Miyagi 2017, Sakai 2017b, Hinoki 2018, Tamadera and Yoshitake 2018, Tamadera et al. 2018, Tamadera and Shigetoh 2019).

#### Notes

New to Iejima Is., these specimens were collected by visual searching and net-sweeping *Macaranga tanarius* (Euphorbiaceae) which is not recorded as a host plant of *C. hastanus* (Figs 3b, 4d, 5b). This buprestid beetle is almost certainly associated with *M. tanarius*, because all specimens were collected only from the plant. In addition, *Mallotus japonicus* (Euphorbiaceae), which has been known as a host plant of this species, was not found in and around each collecting site, as far as we searched.

### Sambus quadricolor quadricolor

Saunders, 1873

40A47963-4E9F-5BCB-AC92-90679F92C937

#### Materials

- **Type status:** Other material. **Occurrence:** recordedBy: Yutaka Tamadera; individualCount: 43; sex: 25 males, 18 females; lifeStage: adult; **Location:** islandGroup: Ryukyus; island: Iejima Island; country: Japan; stateProvince: Okinawa; locality: Mt. Gusuku-yama, Higashie-ue, Ie-son; **Identification:** identifiedBy: Yutaka Tamadera; dateIdentified: 2018; **Event:** eventDate: 06/08/2018; **Record Level:** basisOfRecord: PreservedSpecimen
- **Type status:** Other material. **Occurrence:** recordedBy: Hiraku Yoshitake; individualCount: 24; sex: 9 males, 15 females; lifeStage: adult; **Location:** islandGroup: Ryukyus; island: Iejima Island; country: Japan; stateProvince: Okinawa; locality: Mt. Gusuku-yama, Higashie-ue, Ie-son; **Identification:** identifiedBy: Yutaka Tamadera; dateIdentified: 2018; **Event:** eventDate: 06/08/2018; **Record Level:** basisOfRecord: PreservedSpecimen
- **Type status:** Other material. **Occurrence:** recordedBy: Yutaka Tamadera; individualCount: 7; sex: 4 males, 3 females; lifeStage: adult; **Location:** islandGroup: Ryukyus; island: Iejima Island; country: Japan; stateProvince: Okinawa; locality: Mt. Gusuku-yama, Higashie-ue, Ie-son; **Identification:** identifiedBy: Yutaka Tamadera; dateIdentified: 2018; **Event:** eventDate: 06/09/2018; **Record Level:** basisOfRecord: PreservedSpecimen

#### Distribution

Japan: Honshu (Kanagawa Pref. and Shizuoka Pref.), Kyushu, Sarushima Is. (in Yokosuka-shi), the Ôsumi Isls. (Yakushima Is.), Amami Isls. (Amami-Ôshima Is.) and Okinawa Isls. (Okinawajima Is. and Hamahigajima Is.; Iejima Is. — new record) (Ohmomo and Fukutomi 2013, Kusui and Miyagi 2017).

#### Notes

New to Iejima Is., these specimens were collected by visual searching, net-sweeping and beating *Ficus pumila* which grows on an artificial wall (Figs 3c, 4e, 5c).

### Agrilus okinawensis shiozakii

Tôyama, 1985

F0CD85C7-1A17-5FA7-83A6-B3215CA70853

#### Materials

- **Type status:** Other material. **Occurrence:** recordedBy: Yutaka Tamadera; individualCount: 2; sex: 1 male, 1 female; lifeStage: adult; **Location:** islandGroup: Ryukyus; island: Iejima Island; country: Japan; stateProvince: Okinawa; locality: GI beach, Kawahira, Ie-son; **Identification:** identifiedBy: Yutaka Tamadera; dateIdentified: 2018; **Event:** eventDate: 06/09/2018; **Record Level:** basisOfRecord: PreservedSpecimen
- **Type status:** Other material. **Occurrence:** recordedBy: Yutaka Tamadera; individualCount: 6; sex: 3 males, 3 females; lifeStage: adult; **Location:** islandGroup: Ryukyus; island: Iejima Island; country: Japan; stateProvince: Okinawa; locality: near GI beach, Nishie-mae, Ie-son; **Identification:** identifiedBy: Yutaka Tamadera; dateIdentified: 2018; **Event:** eventDate: 06/09/2018; **Record Level:** basisOfRecord: PreservedSpecimen
- **Type status:** Other material. **Occurrence:** recordedBy: Yutaka Tamadera; individualCount: 6; sex: 1 male, 5 females; lifeStage: adult; **Location:** islandGroup: Ryukyus; island: Iejima Island; country: Japan; stateProvince: Okinawa; locality: near Shôtaiji Temple, Nishie-mae, Ie-son; **Identification:** identifiedBy: Yutaka Tamadera; dateIdentified: 2018; **Event:** eventDate: 06/10/2018; **Record Level:** basisOfRecord: PreservedSpecimen

#### Distribution

Japan: the Amami Isls. (Amami-Ôshima Is.) and Okinawa Isls. (Okinawajima Is., Ikeijima Is. and Kumejima Is.; Iejima Is. — new record) (Ohmomo and Fukutomi 2013, Tamadera et al. 2019).

#### Notes

New to Iejima Is., these specimens were collected by net-sweeping *Pongamia pinnata* (Fabaceae) (Figs 4f, 5d).

## Discussion

By this study, the number of buprestid beetles, known from Iejima Is., has been increased from one to seven species belonging to six genera in three subfamilies. Amongst them, four species, Chrysodema (Marcsikiella) dalmanni, Chrysodema (Chrysodema) lewisii, *Chalcophora japonica oshimana* and *Coraebus hastanus*, are common species and broadly distributed in the Okinawa Isls. (Table 1), since their associated plants are also common and abundant in many islands. With regard to *Chalcophora japonica oshimana*, marine drifts might have played a role in the dispersal of the species from island to island in the Okinawa Isls. (Kurosawa 1974). On the other hand, the remaining three species, *Paratrachys princeps chujoi*, *Sambus quadricolor quadricolor* and *Agrilus okinawensis shiozakii*, are relatively rare and known from only a few islands of the Okinawa Isls. (Table 1), but their associated plants are never rare and broadly distributed. Thus, these buprestids are likely to be distributed in the other adjacent islands.

Generally, larger islands tend to have more species, while smaller islands tend to have fewer species (Whittaker and Fernández-Palacios 2007), but the number of buprestid species presently known from Iejima Is. is less than that from some smaller islands in the Okinawa Isls. (Table 1). Probably due to excessive deforestation in Iejima Is., the buprestid fauna of this island seems to have become considerably poorer than in the past. However, it is still possible that some additional buprestids will be found on Iejima Is., since our field survey was conducted during a short period in the early summer of 2018.

## Supplementary Material

XML Treatment for Paratrachys princeps chujoi

XML Treatment for Chrysodema (Marcsikiella) dalmanni

XML Treatment for Chrysodema (Chrysodema) lewisii

XML Treatment for Chalcophora japonica oshimana

XML Treatment for Coraebus hastanus

XML Treatment for Sambus quadricolor quadricolor

XML Treatment for Agrilus okinawensis shiozakii

## Figures and Tables

**Figure 3a.**
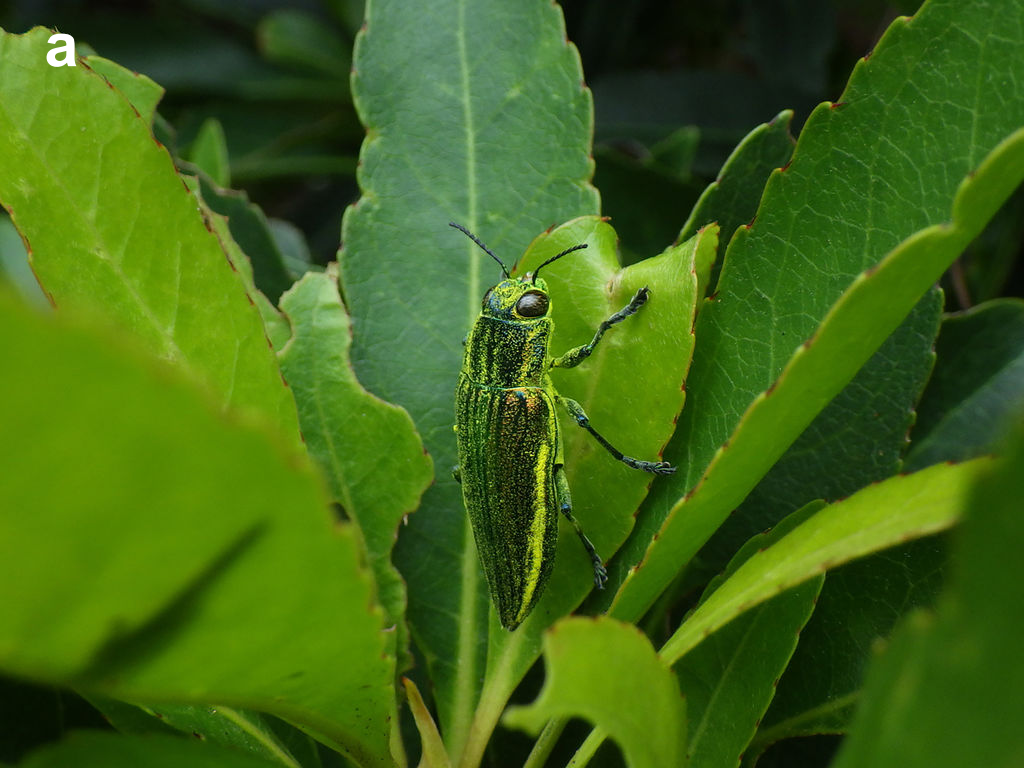
Chrysodema (Chrysodema) lewisii on *Elaeocarpus zollingeri*.

**Figure 3b.**
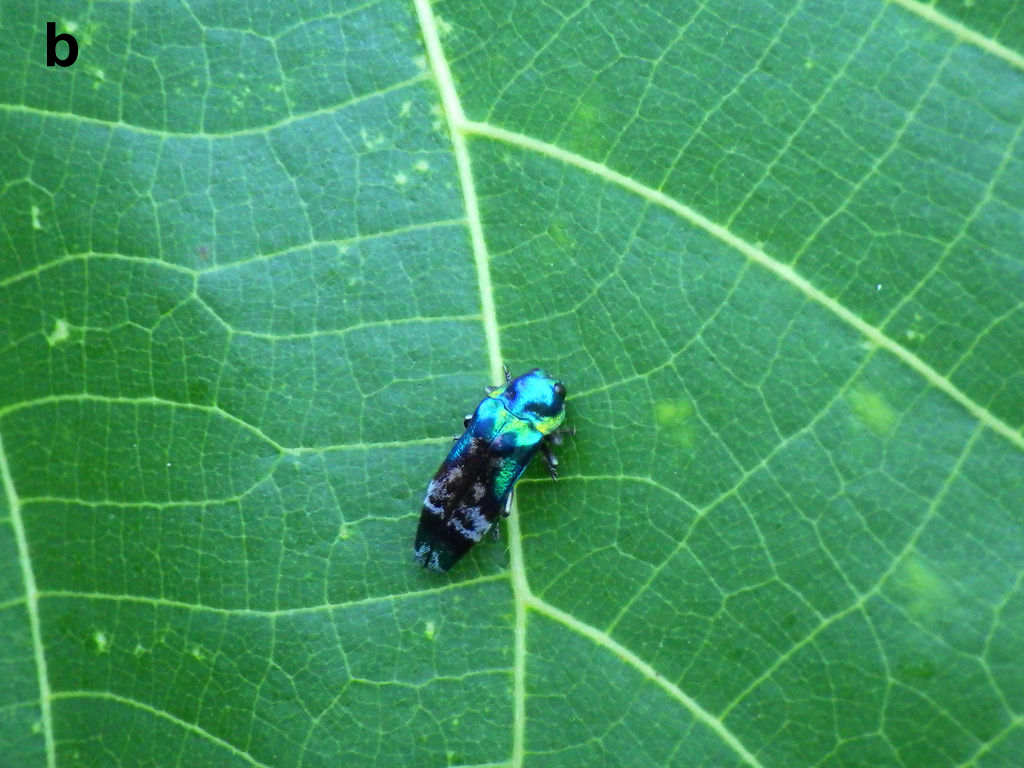
*Coraebus hastanus* on *Macaranga tanarius*.

**Figure 3c.**
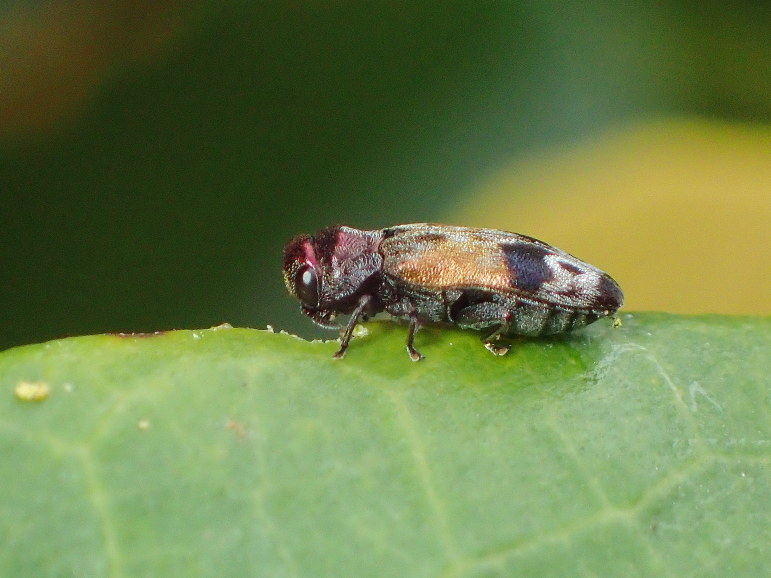
*Sambus quadricolor quadricolor* on *Ficus pumila*.

**Figure 3d.**
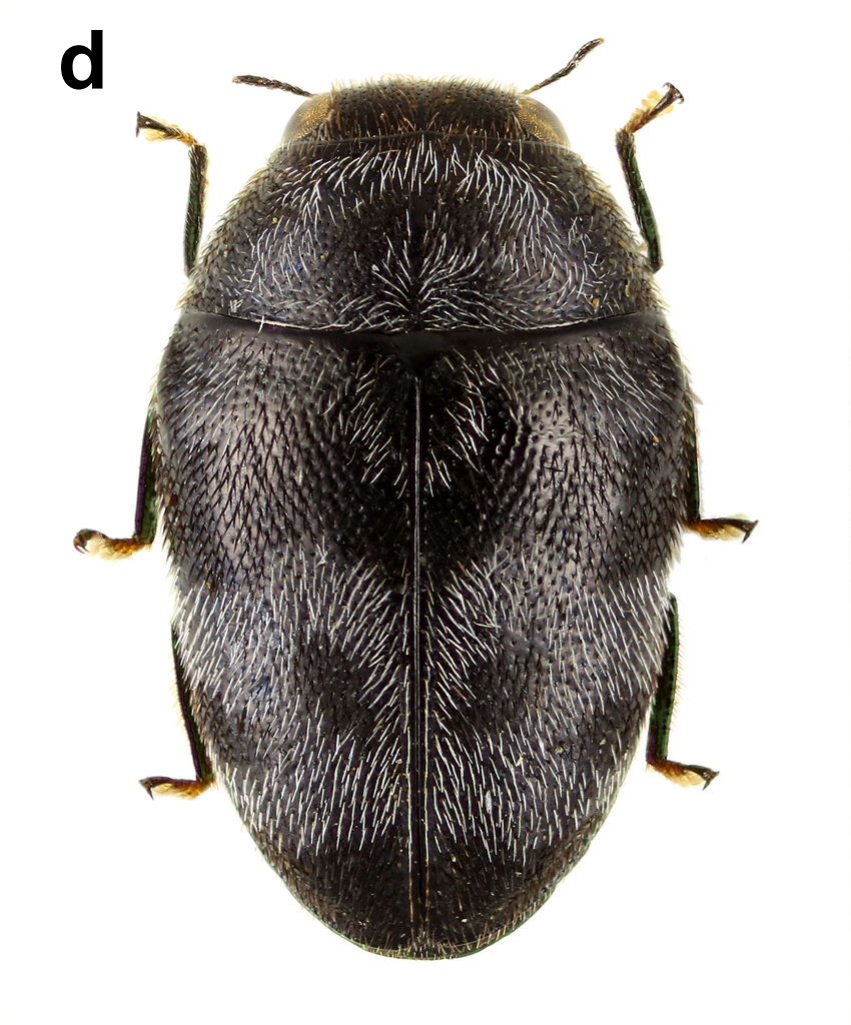
*Paratrachys princeps chujoi*.

**Figure 3e.**
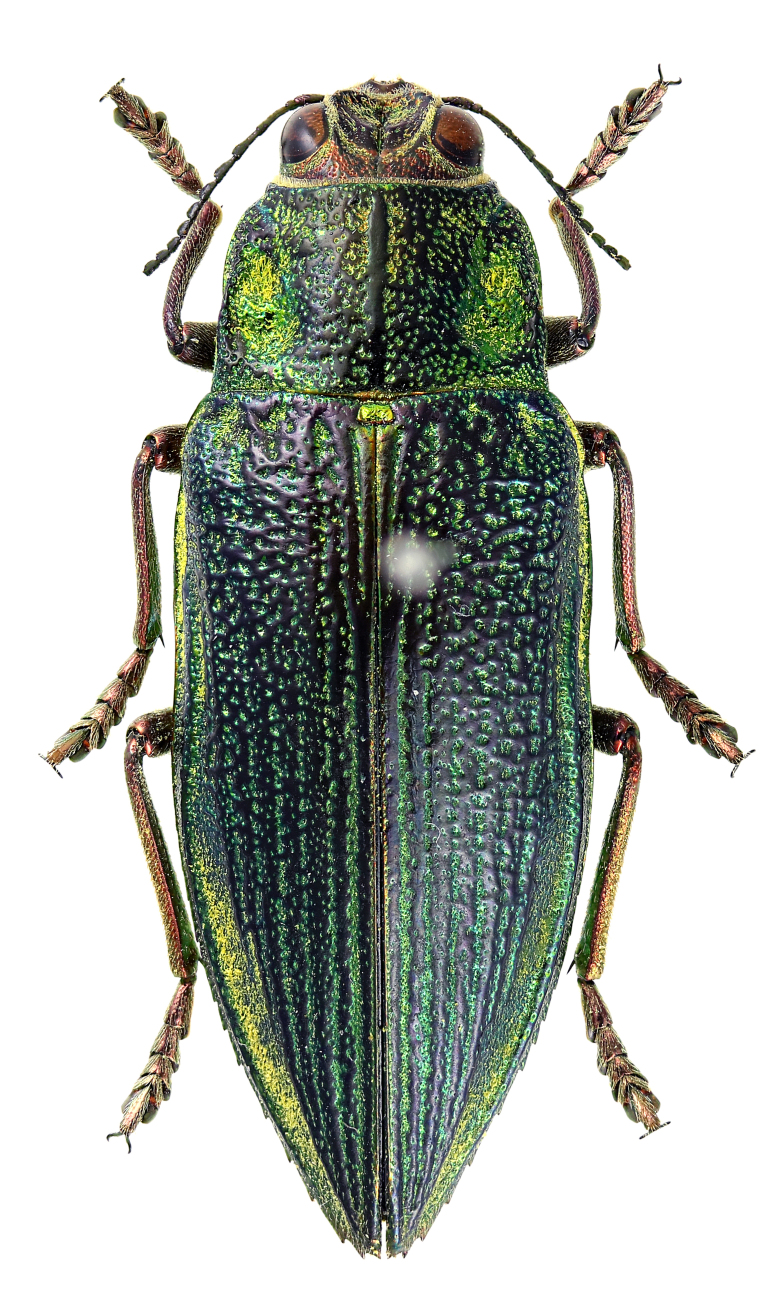
Chrysodema (Marcsikiella) dalmanni.

**Figure 3f.**
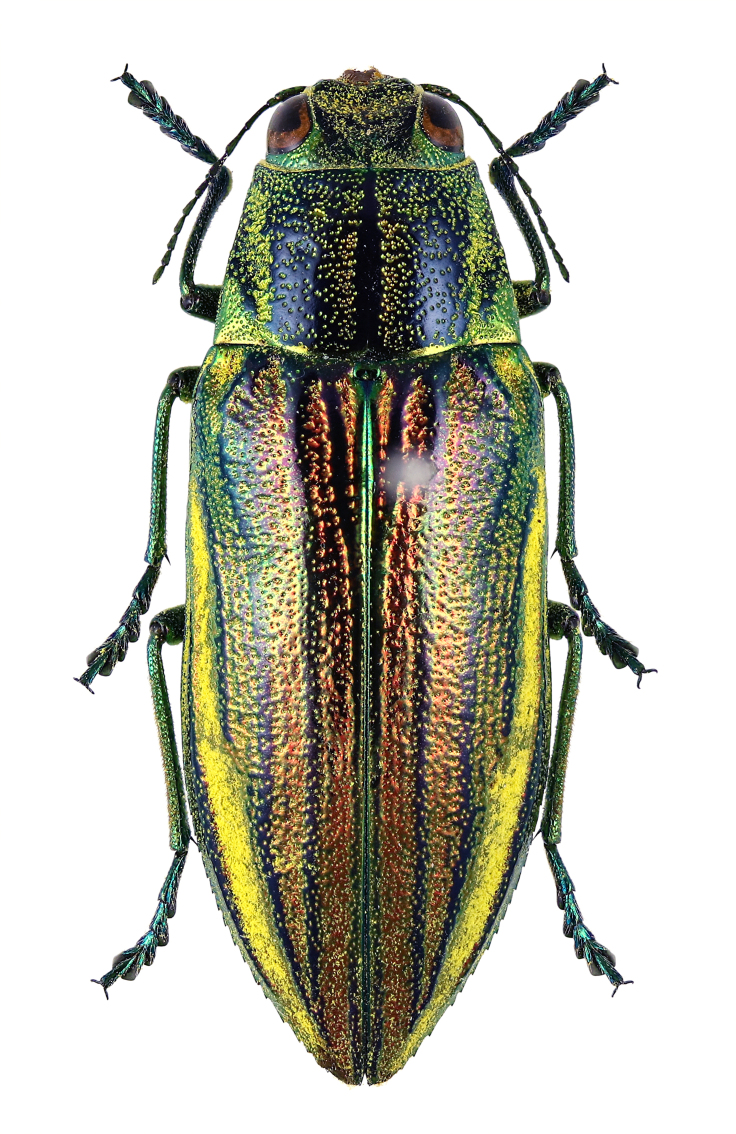
Chrysodema (Chrysodema) lewisii.

**Figure 4a.**
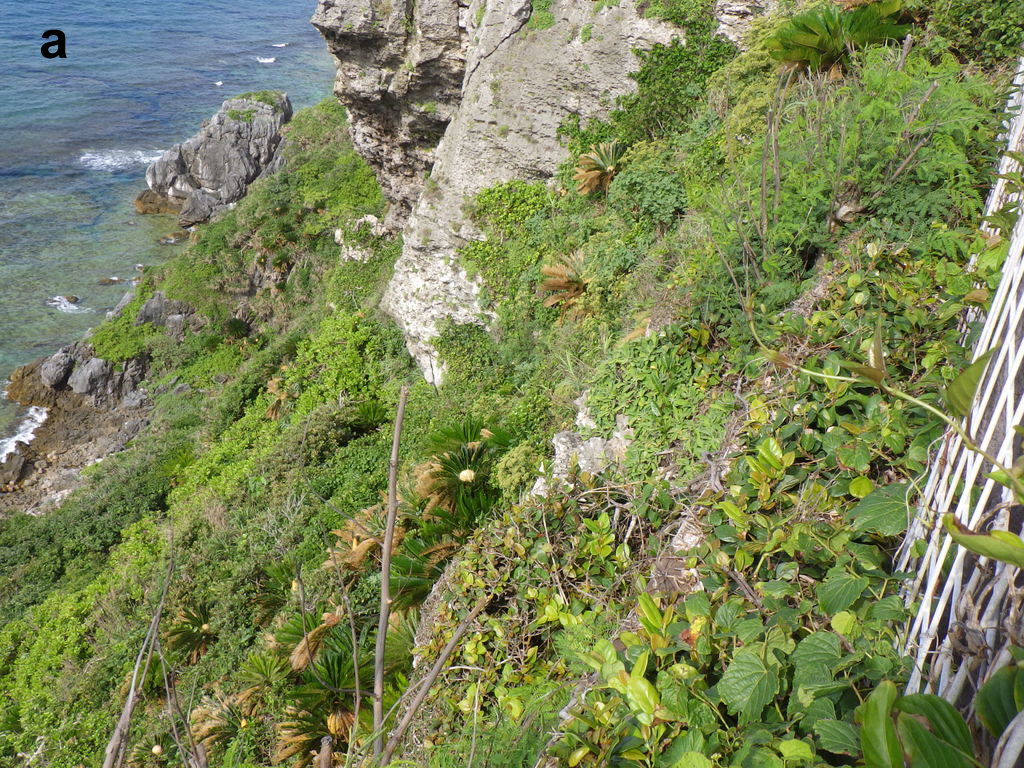
A cliff facing the sea.

**Figure 4b.**
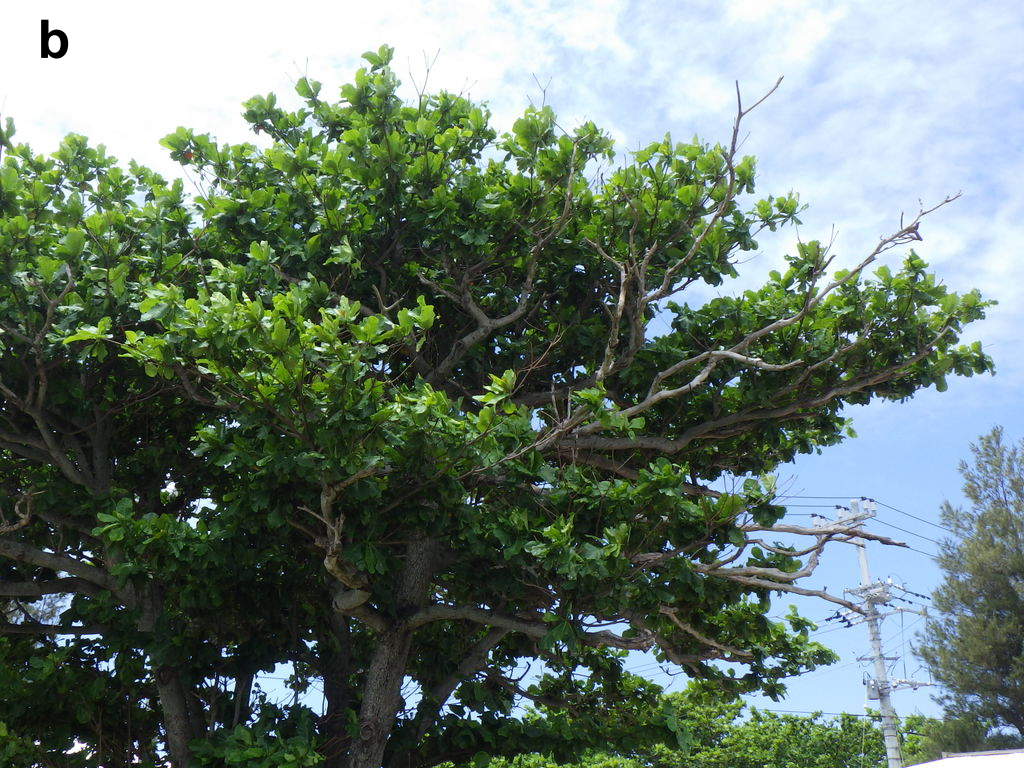
*Terminalia catappa* infested by Chrysodema (Marcsikiella) dalmanni.

**Figure 4c.**
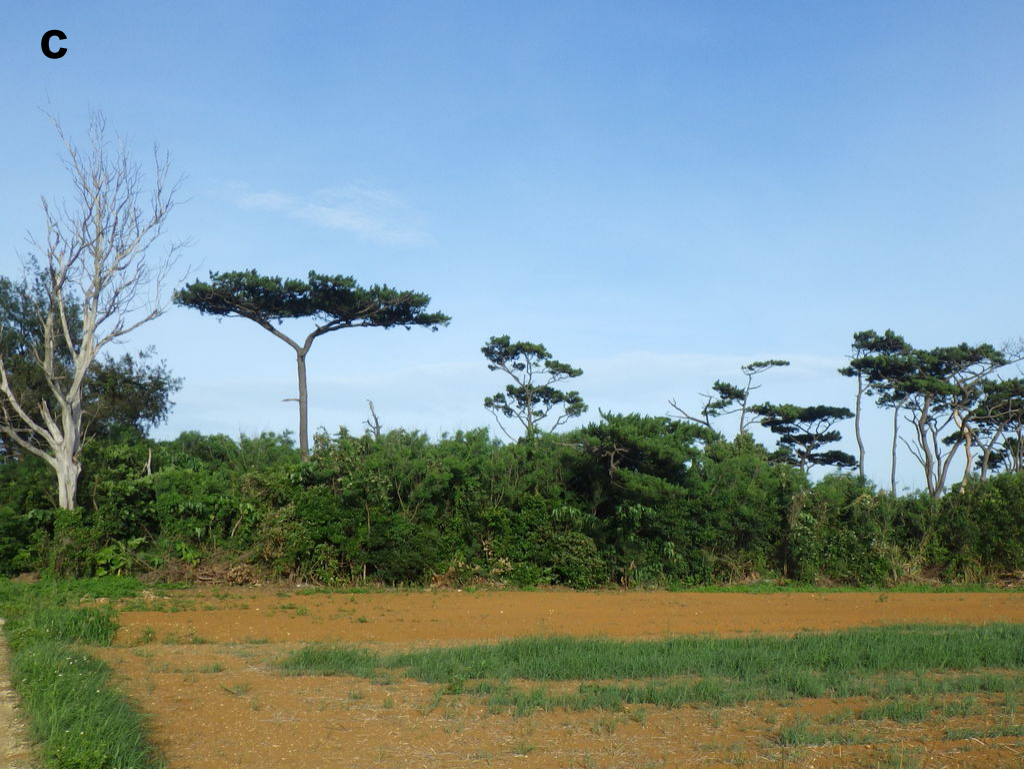
A small forest left in cropland.

**Figure 4d.**
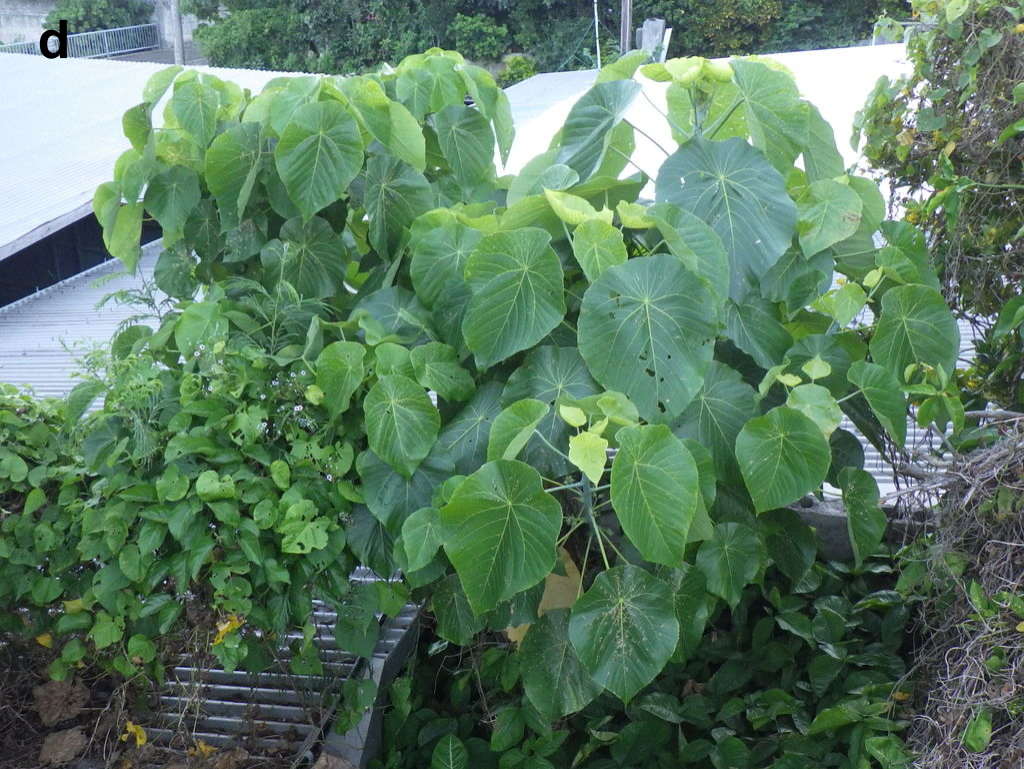
*Macaranga tanarius* infested by adult *Coraebus hastanus*.

**Figure 4e.**
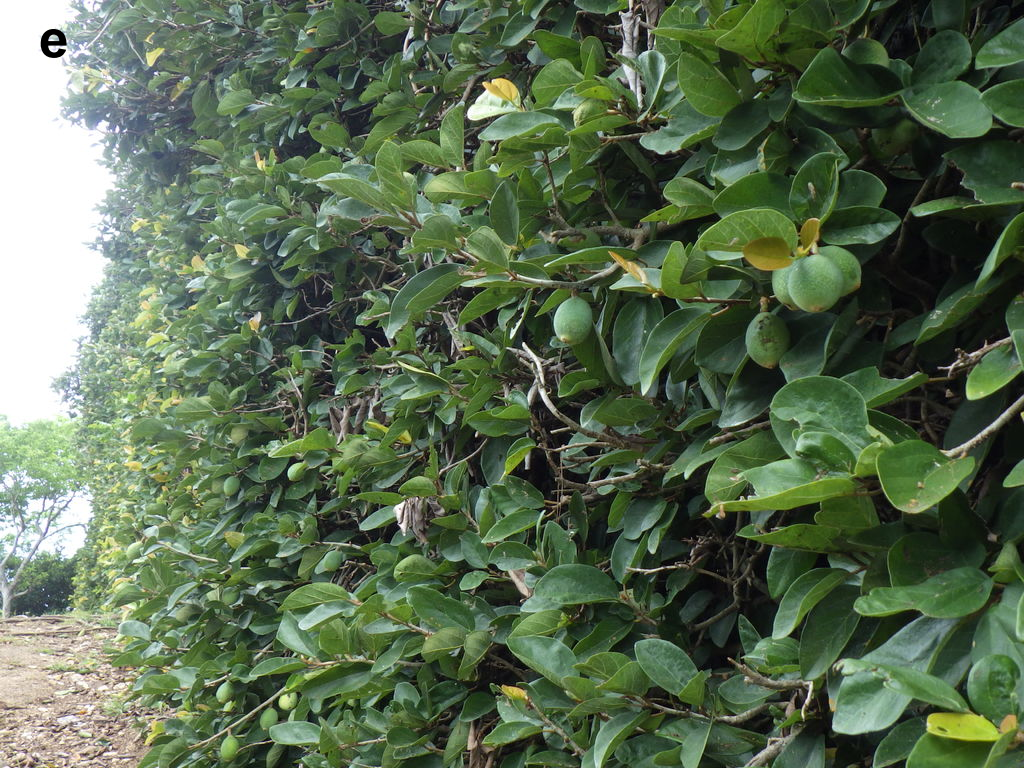
*Ficus pumila* on an artificial wall.

**Figure 4f.**
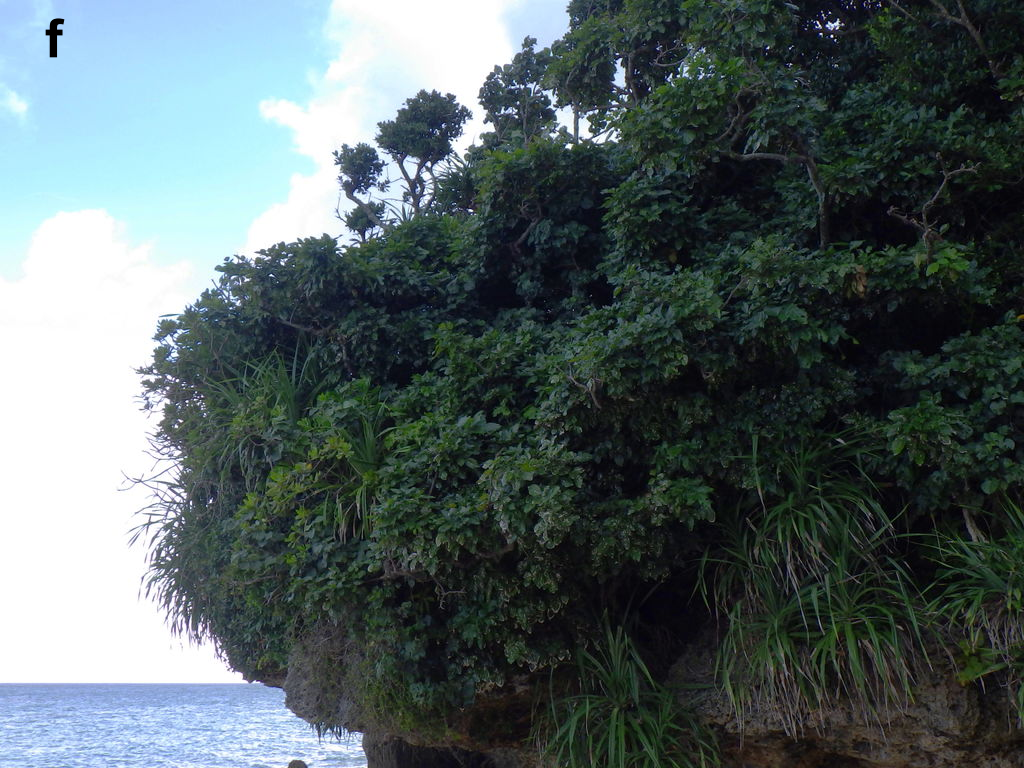
*Pongamia pinnata* in a coastal forest.

**Figure 5a.**
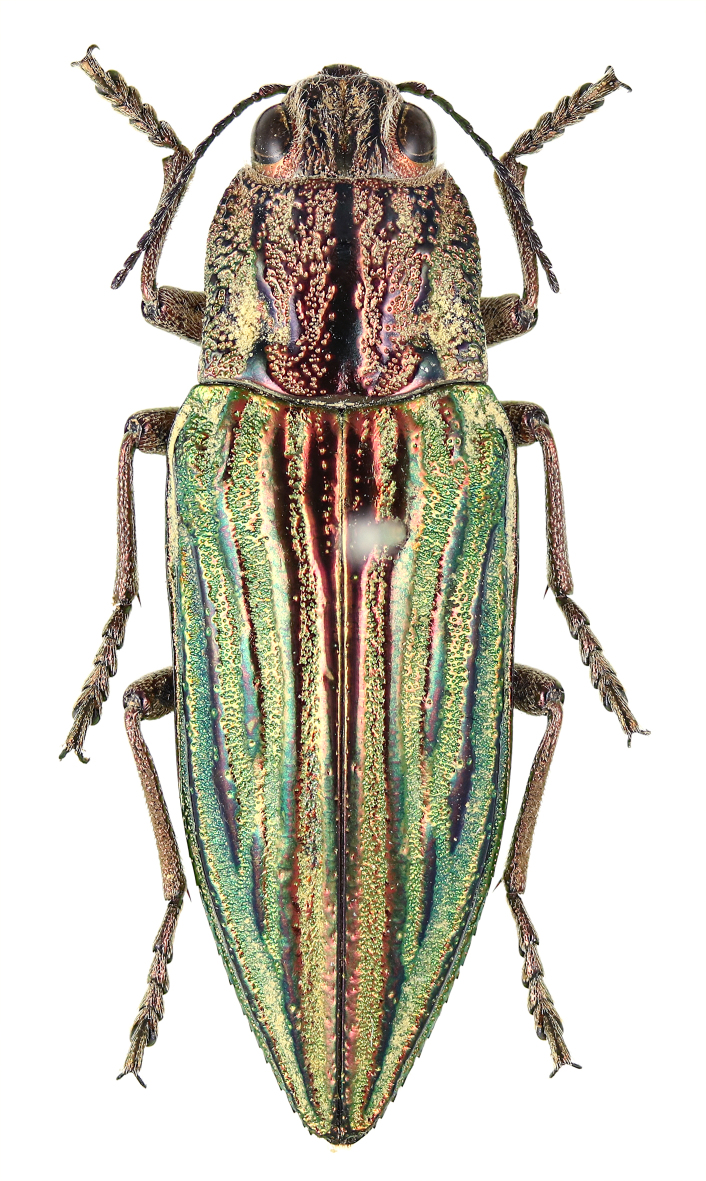
*Chalcophora japonica oshimana*.

**Figure 5b.**
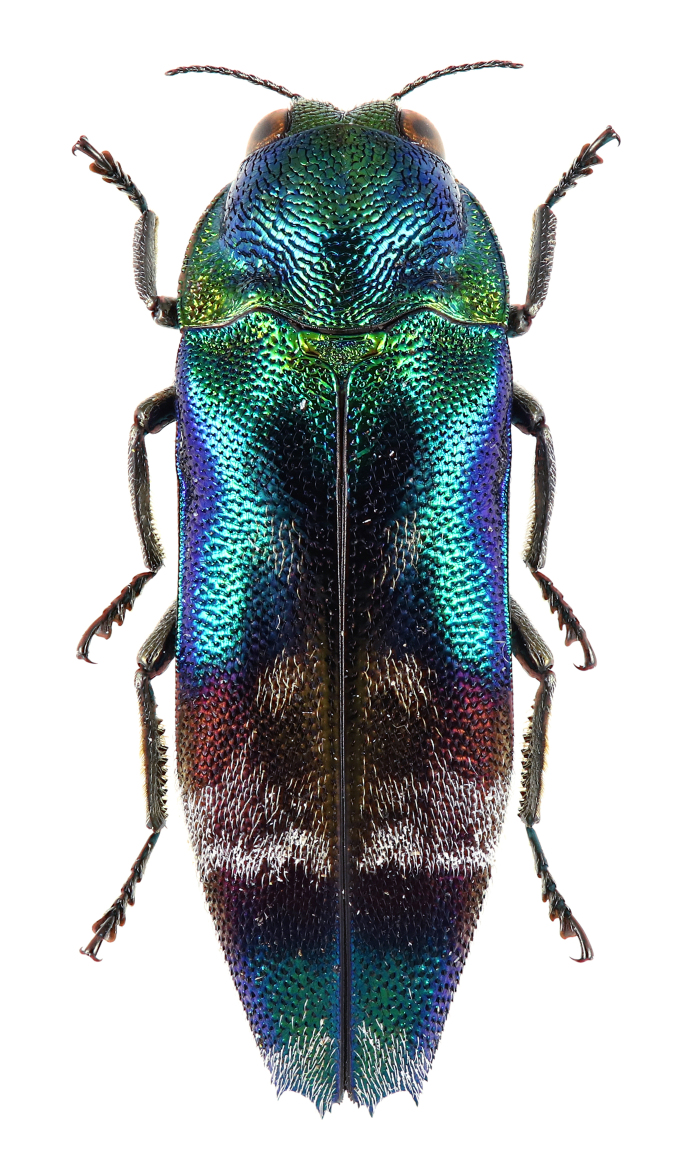
*Coraebus hastanus*.

**Figure 5c.**
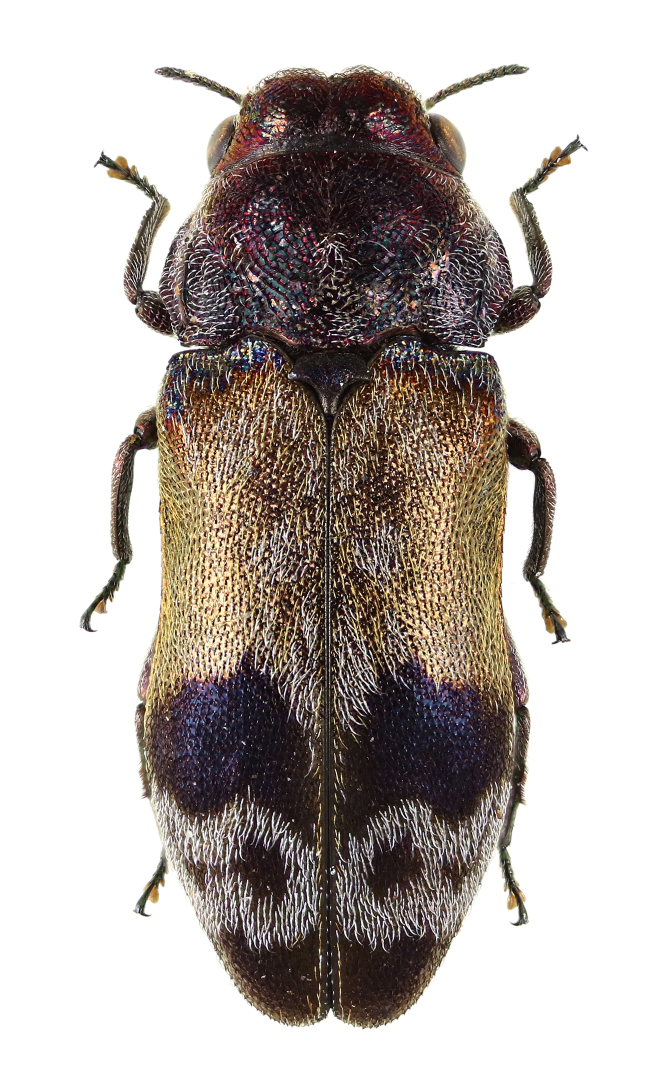
*Sambus quadricolor quadricolor*.

**Figure 5d.**
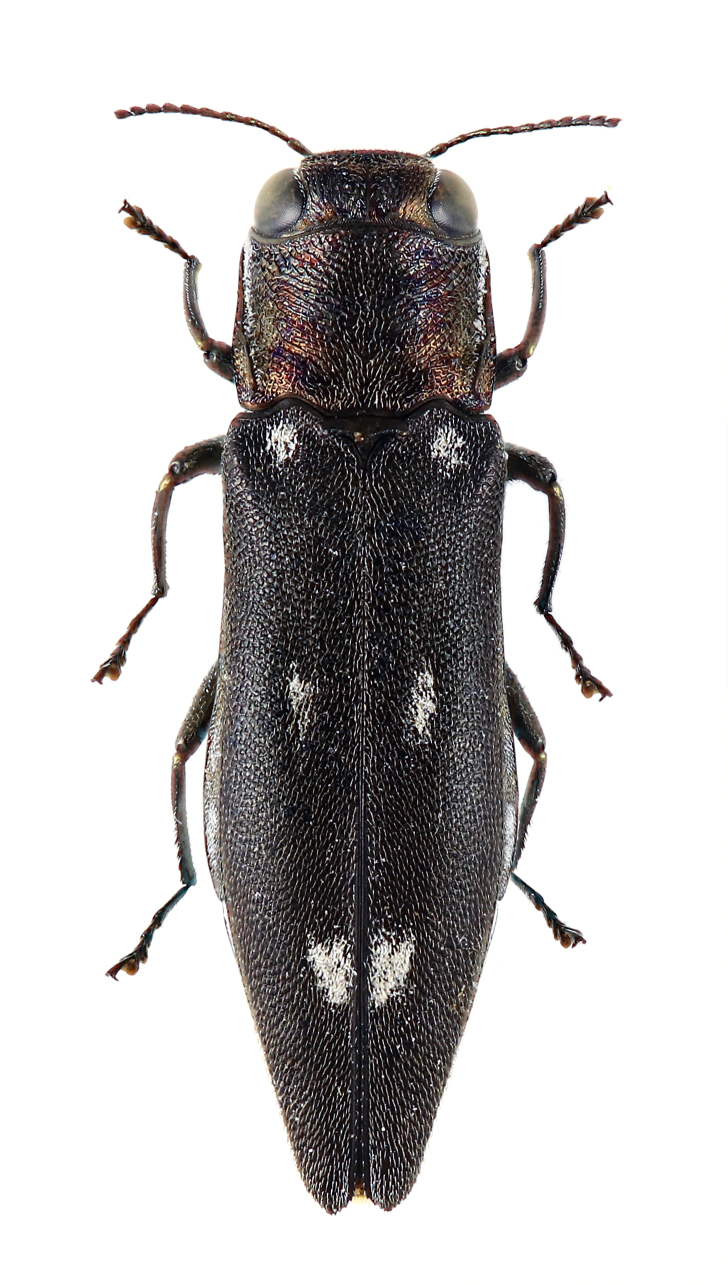
*Agrilus okinawensis shiozakii*.

**Table 1.** Commonality of buprestid species between Iejima Island and some other islands of the Okinawa Islands (area (km^2^) of and the number of known species from each island are indicated in parentheses).

Species	Islands					
	Iejima (22.8; 7)	Okinawajima (1,207; 44)	Kumejima (59.5; 13)	Iheyajima (20.7; 8)	Tokashikijima (15.3; 8)	Zamamijima (6.7; 5)
*Paratrachys princeps chujoi*	+	+	-	-	-	-
Chrysodema (Marcsikiella) dalmanni	+	+	+	+	+	+
Chrysodema (Chrysodema) lewisii	+	+	+	+	+	+
*Chalcophora japonica oshimana*	+	+	+	+	+	+
*Coraebus hastanus*	+	+	+	+	-	-
*Sambus quadricolor quadricolor*	+	+	-	-	-	-
*Agrilus okinawensis shiozakii*	+	+	+	-	-	-
